# Severe maternal outcomes and quality of care at district hospitals in Rwanda– a multicentre prospective case-control study

**DOI:** 10.1186/s12884-017-1581-4

**Published:** 2017-11-25

**Authors:** Felix Sayinzoga, Leon Bijlmakers, Koos van der Velden, Jeroen van Dillen

**Affiliations:** 1 Maternal, Child and Community Health Division, Rwanda Ministry of Health, Rwanda Biomedical Center, PO Box 84, Kigali, Rwanda; 2 Department of Health Evidence, Radboud University Medical Centre Nijmegen, Nijmegen, The Netherlands; 3 Department of Primary and Community Care, Radboud University Medical Centre Nijmegen, Nijmegen, The Netherlands; 4 Department of Obstetrics and Gynaecology, Radboud University Medical Centre Nijmegen, Nijmegen, The Netherlands

**Keywords:** Severe maternal outcome, Maternal near miss, Obstetrics, Quality of care

## Abstract

**Background:**

Despite a significant decrease in maternal mortality in the last decade, Rwanda needs further progress in order to achieve Sustainable Development Goals (SDG)3 which addresses among others maternal mortality. Analysis of severe maternal outcomes (SMO) was performed to identify their characteristics, causes and contributory factors, using standard indicators for quality of care.

**Methods:**

A prospective case-control study was conducted for which data were collected between November 2015 and April 2016 in four rural district hospitals. The occurrence of SMO with near miss incidence ratios was established, followed by an analysis of the characteristics, clinical outcomes, causes and contributory factors.

**Results:**

The SMO incidence ratio was 38.4 per 1000 live births (95% CI 33.4–43.4) and the maternal near-miss incidence ratio was 36 per 1000 live births (95% CI 31.1–40.9). The leading causes of SMO were postpartum haemorrhage (23.4%), uterine rupture (22.9%), abortion related complications (16.8%), malaria (13.6%) and hypertensive disorders (8.9%). The case fatality rate was high for women with hypertensive disorders (10.5%; CI 3.3–24.3) and severe postpartum haemorrhage (8%; CI 0.5–15.5). Stillbirth (OR = 181.7; CI 43.5–757.9) and length of stay at the hospital (OR = 7.9; CI 4.5–13.8) were strongly associated with severe outcomes.

**Conclusions:**

Despite the use of life saving interventions, SMO are frequent. Mortality index was found to be low at the level of district hospitals. SMO were associated with long stay at the hospital and stillbirth. There is a need for improvement of quality of care, referral practices and certain types of infrastructure, especially blood banks, which would ensure truly comprehensive emergency obstetric care and reduce the occurrence of SMO.

**Electronic supplementary material:**

The online version of this article (10.1186/s12884-017-1581-4) contains supplementary material, which is available to authorized users.

## Background

Globally, the Maternal Mortality Ratio (MMR) has fallen by nearly 44% over the past 25 years, to an estimated 216 maternal deaths per 100,000 live births in 2015. Despite this global decline, the magnitude of the reduction differed substantially between regions, with low and middle-income countries accounting for approximately 99% of global maternal deaths and sub-Saharan Africa alone accounting for roughly 66% [1]. Rwanda is one of only four countries that have achieved the Millennium Development Goals 4 and 5 (MDGs 4 and 5) [2]. According to the Rwanda Demographic Health Survey (RDHS) 2014–2015, the MMR decreased from 1071 in 2000 to 210 in 2015, while the percentage of institutional deliveries increased from 27% to 91% over the same period [3, 4]. However, a fast increase of deliveries in health facilities may compromise the quality of care that mothers receive, especially in primary care facilities [5].

The Sustainable Development Goals (SDGs) now call for an acceleration of progress in order to achieve a global MMR of 70 maternal deaths per 100,000 live births or less by 2030 [1]. Rwanda has demonstrated a strong political will to improve maternal and newborn health. One of the measures taken to achieve this was the introduction in 2009 of maternal death audits (MDA) on a routine basis nationwide [6]. However, since maternal mortality reveals only the tip of the iceberg, several countries have initiated maternal near-miss audits [7]. Rwanda might be able to further improve its performance by reviewing the circumstances that led to maternal near miss events where the women survived. By evaluating such cases much can be learnt about the processes in place and systemic deficiencies that cause failure to deal with maternal morbidities. For this purpose WHO recommends the near-miss approach for maternal health [8].

Few studies have been done on maternal near-miss in Rwanda. They were conducted in tertiary hospitals situated in Kigali [9–11], or in provincial referral hospitals that are better equipped than district hospitals in terms of infrastructure (e.g. intensive care units, ICU) and human resources [12]. District hospitals in Rwanda normally handle only cases that are referred by health centres, because of high-risk pregnancy or the occurrence of complications. Approximately 80% of all deliveries occur at the level of health centres [13]. Due to this risk selection system, near-miss data from tertiary or provincial hospitals do not reflect common practice at lower level health facilities. In addition, most studies on maternal near-miss are descriptive, based on case series. We conducted a multicentre, case-control study of severe maternal outcome (SMO) and women without SMO at district hospital level. Apart from the health outcomes, we also assessed process indicators, using standard indicators for quality of care.

## Methods

### Study design

This was a prospective case-control study, for which data were collected between November 2015 and April 2016 in four rural district hospitals. The four districts were purposively selected based on their performance on a selected set of key maternal and child health indicators from the national Health Management Information System (HMIS) in 2013: two of them were good performers (Bugesera and Rwamagana districts) and two performed poorly (Nyagatare and Gicumbi) regarding maternal health. The four district hospitals serve a total population of more than 1.5 million people (approximately 10% of the country’s total population) and in total they have 76 rural health centers in their catchment areas.

### Definition

A woman with a severe maternal outcome (SMO) could be either a maternal near-miss case or a woman who actually died [8].

Maternal near-miss (MNM) is defined as ‘a woman who nearly died, but survived a complication that occurred during pregnancy, childbirth or within 42 days of termination of pregnancy.’ [7].

### Sampling

All cases fulfilling the criteria of severe maternal outcome during the study period were included in the study. The WHO criteria adapted in the Haydom study (listed in the Additional file 1: Table S1) in Tanzania were applied [14, 15]. Cases were identified by health providers who were on duty at the time of admission or who noticed a deterioration in the woman’s condition during her stay at the hospital. Controls were selected from in-patient women who had given birth or were admitted for pregnancy complications and who did not have a severe maternal outcome within 48 h of the occurrence of the case. At least one of the following characteristics similar to the case was used to select the control: age, parity, gestational age and mode of delivery. The number of near-miss cases required was estimated using the sampsi_mcc function in Stata for sample size calculation [16]. The parameters for the calculation were: power of 80% at 5% statistical significance level and odds ratio of 2. The minimum sample size required was 120 near-miss cases and two controls per case, giving a total of 240 controls.

### Data collection

Relevant data for cases and controls were extracted from patient medical files (personal characteristics, clinical information) and entered into a template developed from the WHO near-miss approach for maternal health guide [7]. Missing information, if any, was obtained mostly from the health centre that had referred the case; especially information about the patient’s arrival time at the clinic, at what time the ambulance was called and when it actually arrived, and the medical status of the mother and the fetus/infant prior to referral. Additionally, data were collected on maternal and neonatal outcomes and on particular interventions that had been undertaken to prevent and/or manage complications (for example use of oxytocin for the prevention and treatment of postpartum haemorrhage among cases and controls who gave birth at hospital; use of magnesium sulfate for treatment of eclampsia; use of antibiotics for prophylaxis and treatment of sepsis). The inclusion criteria were displayed in the maternity departments of the four hospitals as a reminder to health staff who were required to identify cases and controls. Data collection forms were made available at the place where patient registers were kept, in order to facilitate completion by health staff who had identified patients with SMO.

The head of the maternity served as the focal point for the study and was trained along with several other staff from each hospital. He/she was also responsible for collecting any missing information from the health centre that had referred the patient.

Every 4 weeks, the principal investigator (FS) visited the hospitals for verification of the completed forms with the maternity team: this involved checks on the correct application of inclusion/exclusion criteria and checks for completeness and consistency of data. The forms were reviewed case by case with the respective hospital teams.

### Data analysis

Data were entered into an Excel template and then reviewed for inconsistencies. Statistical analysis was performed using SPSS Statistics, version 23 (SPSS Inc. Chicago, Illinois). Univariate analysis was carried out to characterize the SMO cases and controls in terms of demographic and clinical variables and the underlying causes. Statistical differences between SMO cases and controls were compared using chi-square test. Outcome indicators were calculated as proposed by WHO [8], using the total number of live births during the study period and the total number of maternal near-miss and maternal death in the same period. All descriptive data, including the identified underlying causes, are reported both as absolute numbers (n) and frequencies (%).

As for process indicators, the principal researcher in collaboration with the local maternity team identified the target population for each of the specific interventions of interest, on the basis of which the proportion of the target population that actually received the recommended intervention was calculated. High proportions of women receiving appropriate interventions indicate better quality of care. Crude (cOR) and adjusted (aOR) odds ratios (including 95% confidence intervals) were calculated for predictive factors using logistic regression. Only factors that were statistically significant in univariate analysis were considered for logistic regression. Associated factors were also examined for statistical significance, using chi-square tests and bivariate logistic regression. The dependent variable was severe maternal outcome and the independent factors were the status of the infant at birth and the duration of admission.

## Results

### Characteristics of women with SMO and controls

During the 5 months data collection period, 5577 live births were recorded in the four district hospitals. Out of these, 214 cases of severe maternal outcomes were identified, of which 201 maternal near-miss cases and 13 maternal deaths. A total of 428 controls were selected and included in the study. In total the study population comprised 642 women.

The comparison of SMO cases and controls shows statistically significant differences in age, marital status and profession (Table 1). Seven-and-a-half percent of the cases were younger than 20 years (3.5% among controls) and 28.4% older than 35 years (12.6% among controls). Unmarried and unemployed women are seen more among the cases than in the control group (15.0% versus 5.6%, and 4.2% versus 0.2%, respectively). The two groups differ significantly with respect to some clinical characteristics of pregnancy, such as parity, gravidity, number of antenatal consultation (ANC) and gestational age. The proportion of SMO was high among women who did not attend any ANC (27.4%), and in women with gestational age less than 36 weeks (21.7% for <24 weeks and 18.5% for 24–36 weeks). The proportion of stillbirths among the cases was very high (46.1% versus 0.5% in the control group). Similarly, cases were admitted for a longer period (70% for 4 days or more) than controls (31.7%; figures not shown in the table). There were no statistically significant differences between the two groups regarding educational level, medical insurance status, previous abortion, previous caesarean section and mode of delivery.

**Table 1:** Socio-demographic and clinical characteristics of the SMO cases and controls

	Cases (%)	Controls (%)	Total	Chi-square	*P*-value
Age					
< 20	16 (7.5)	15 (3.5)	31 (4.8)	22.02	0.000
20–35	145 (67.8)	359 (83.9)	504 (78.5)		
> 35	53 (28.4)	54 (12.6)	107 (16.7)		
Marital status^a^ (*N* = 630)					
Married	182 (85.0)	402 (94.4)	584 (91.3)	15.49	0.000
Unmarried	32 (15.0)	24 (5.6)	56 (8.8)		
Profession^a^ (*N* = 641)					
Farmer	200 (93.5)	423 (99.1)	623 (97.2)	17.92	0.000
Other profession	5 (2.3)	3 (0.7)	8 (1.2)		
Unemployed	9 (4.2)	1 (0.2)	10 (1.6)		
Education level^a^ (*N* = 637)					
Never been to school	15 (7.1)	21 (4.9)	36 (5.7)	7.36	0.25
Primary education	174 (82.5)	327 (76.8)	501 (78.6)		
Secondary and higher	22 (10.4)	78 (18.3)	100 (15.7)		
Medical Insurance					
No	13 (6.1)	38 (8.9)	51(7.9)	1.53	0.216
Yes	201 (93.9)	390 (91.1)	591 (92.1)		
Clinical characteristics					
Parity					
0	60 (28.0)	179 (41.8)	239 (37.2)	15.18	0.002
1	41 (19.2)	78 (18.2)	119 (18.5)		
2 to 4	84 (39.3)	140 (32.7)	224 (34.9)		
≥ 5	29 (13.6)	31 (7.2)	60 (9.3)		
Gravidity					
1	65 (30.4)	181 (42.3)	246 (38.3)	13.25	0.001
2 to 4	105 (49.1)	197 (46.0)	302 (47.0)		
≥ 5	44 (20.6)	50 (11.7)	94 (14.6)		
Previous C/S					
No	152 (71.0)	325 (75.9)	477 (74.3)	1.80	0.18
Yes	62 (29.0)	103 (24.1)	165 (25.7)		
Previous abortion					
No	204 (95.3)	401 (93.7)	605 (94.2)	0.70	0.402
Yes	10 (4.7)	27 (6.3)	37 (5.8)		
ANC^a^ (*N* = 626)					
0	55 (27.4)	44 (10.4)	99 (15.8)	31.91	0.000
1	27 (13.4)	50 (11.8)	77 (12.3)		
2 to 3	95 (47.3)	263 (61.9)	358 (57.2)		
≥ 4	24 (11.9)	68 (16)	92 (14.7)		
Gestational age^a^ (*N* = 594)					
< 24 weeks	40 (21.7)	1 (0.2)	41 (6.9)	179.67	0.000
24–36 weeks	34 (18.5)	1 (0.2)	35 (5.9)		
≥ 37 weeks	110 (59.8)	408 (99.5)	518 (87.2)		
Outcomes					
Baby’s condition at birth^a^ (*N* = 568)					
Alive (501)	76 (53.9)	425 (99.5)	501 (88.2)	212.13	0.000
Still birth (67)	65 (46.1)	2 (0.5)	67 (11.8)		
Mode of delivery^a^ (*N* = 532)					
Vaginal delivery (*n* = 274)	44 (45.8)	230 (53.9)	274 (52.4)	2.03	0.155
Caesarean section (*n* = 249)	52 (54.2)	197 (46.1)	249 (47.6)		
Length of hospital stay^a^ (N = 641)					
0 to 1 day	17 (8)	140 (32.7)	157 (24.5)	104.97	0.000
2 to 3 days	47 (22.1)	152 (35.5)	199 (31.0)		
4 to 7 days	126 (59.2)	132 (30.8)	258 (40.2)		
More than 7 days	23 (10.8)	4 (0.9)	27 (4.2)		

^**a**^ Missing information for some cases

### Outcome indicators

The severe maternal outcome incidence ratio was 38.4 per 1000 live births (95% CI 33.4–43.4) and the maternal near-miss incidence ratio was 36.0 per 1000 live births (95% CI 31.1–40.9) (Table 2). For every maternal death there were 15.5 near-miss cases. The hospital-based MMR was 233 per 100,000 live births (95% CI 110–360), with a mortality index (MI) of 6.1% (95% CI 2.9–9.3).

**Table 2:** Maternal outcome indicators in four district hospitals in Rwanda

Live births^a^	5577
Severe maternal outcome indicators	
Women with maternal near-miss (MNM)^b^	201
Maternal death (MD)^c^	13
Women with severe maternal outcomes (SMO)^d^	214
Overall near-miss indicators	
Severe maternal outcome ratio (SMOR) per 1000 live births^e^	38.4 (33.4–43.4)
Maternal near-miss incidence ratio per 1000 live births^f^	36.0 (31.1–40.9)
Maternal near-miss mortality ratio^g^	15.5
Maternal mortality ratio per 100,000 live births^h^	233 (110–360)
Mortality index (%)^i^	6.1 (2.9–9.3)
Hospital access indicators	
Women with SMO at arrival or within 12 h of hospital arrival^k^	188
Proportion of SMO at arrival or within 12 h of hospital arrival (%)^l^	87.9 (83.5–92.3)
Women with SMO at arrival or within 12 h of hospital arrival and referred from HC^m^	177
Proportion of SMO at arrival or within 12 h of hospital arrival referred from HC (%)^n^	94.1 (90.7–97.5)
SMO at arrival or within 12 h of hospital arrival who died^o^	9
SMO at arrival or within 12 h of hospital arrival mortality index (%)^p^	4.8 (1.7–7.9)
Intra hospital care indicators	
Women who developed SMO more than 12 h after hospital arrival (intra hospital SMO)^q^	26
Intra hospital SMO rate (per 1000 live births)^r^	4.7 (2.9–6.5)
Women with SMO developed after 12 h of hospital arrival who died^s^	4
Intra hospital mortality index (%)^t^	15.4 (1.5–29.3)

^a^Live birth (LB): the complete expulsion or extraction from its mother of a product of conception, irrespective of the duration of the pregnancy, which, after such separation, breathes or shows any other evidence of life. Each product of such a birth is considered live born

^b^Number of women with maternal near-miss

^c^Number of maternal death

^d^Women with severe maternal outcome (SMO) the sum of maternal near-miss and maternal deaths. d = (b + c)

^e^Severe maternal outcome ratio (SMOR): the number of women with life threatening conditions per 1000 live births. e = (d/a)*1000LB

^f^MNM incidence ratio: the number of maternal near-miss cases per 1000 live births. [MNM IR = MNM/LB]. f = (b/a)*1000LB

^g^Maternal near-miss mortality ratio: the proportion between maternal near-miss cases and maternal deaths. g = (b/c)

^h^Maternal mortality ratio per 100,000 live births

^i^Case fatality rate: the number of maternal deaths divided by the number of women with SMO, expressed as a percentage. i = (c/d)*100

^k^Number of women with SMO at arrival or within 12 h of hospital arrival

^l^Proportion SMO at arrival or within 12 h among all SMO: the number of SMO who are ill on arrival or within 12 h divided by the total number of SMO. l = (k/d)*100

^m^Number of women with SMO at arrival or within 12 h of hospital arrival and referred from HC

^n^Proportion of SMO at arrival or within 12 h coming from other health facilities: the number of SMO who are ill on arrival or within 12 h and coming from a health center divided by the total number of SMO at arrival or within 12 h. n = (m/k)*100

^o^Number of SMO at arrival or within 12 h of hospital arrival who died

^p^SMO at arrival or within 12 h mortality index: the maternal deaths within 12 h after arrival divided by the number of women with SMO who were ill on arrival or within 12 h, expressed as percentage. p = (o/k)*100

^q^Number of Women who developed SMO more than 12 h after hospital arrival (intra hospital SMO)

^r^ Intra hospital SMO rate (per 1000 live births): the number of women with SMO who developed these life-threatening conditions after 12 h in the hospital per 1000 live births. r = (q/a)*1000LB

^s^Number of women who developed SMO more than 12 h after hospital arrival

^t^Intra hospital mortality index: the number of maternal deaths who were not ill on arrival or within 12 h, divided by the number of women with SMO who were not ill on arrival or within 12 h, expressed as a percentage. t = (s/q)*100

Of the 214 SMO cases (near-miss and maternal deaths combined), 188 (87.9%; CI 83.5–92.3) presented the life-threatening condition on arrival or within the first 12 h of hospital admission; 94.1% (CI 90.7–97.5) of these cases were referred from health centers. Death within 12 h occurred in 9 women (4.8%; 95% CI 1.7–7.9;) of 188 women who were ill on arrival or within 12 h. Twenty-six women developed life-threatening conditions in the hospital after 12 h of admission and four of them (15.4%; 95% CI 1.5–29.3) died. Almost all patients (97.8%) were referred from a health center: only 2.2% of women came straight to the district hospital.

### Predictive and associated factors

In multivariate logistic regression analysis (Table 3), only marital status (unmarried; aOR = 3.53; CI 1.43–8.71) and gestational age (<24 weeks: aOR = 190.15; CI 22.62–1598.87; and 24–36 weeks: aOR = 167.48; CI 21.94–1278.32) remained as predictive factors for SMO. Stillbirth (OR = 181.74; CI 43.47–757.99) and length of stay at the hospital (OR = 7.86; CI 4.49–13.76) were strongly associated with severe outcomes.

**Table 3:** Multivariate analysis of the predictive factors for SMO

	crude OR (95%CI)	adjusted OR (95%CI)
Age (*N* = 642)		
< 20	**2.45 (1.18–5.1)**	1.20 (0.37–3.86)
20–35	1	1
> 35	**1.47 (0.99–2.15)**	1.63 (0.99–2.68)
Marital status (*N* = 640)		
Married	1	1
Unmarried	**2.95 (1.69–5.14)**	**3.53 (1.43–8.71)**
Profession (N = 641)		
Farmer	1	1
Other profession	**3.52 (0.83–14.89)**	4.33 (0.69–27.37)
Unimployed	**19.04 (2.39–151.28)**	0.68 (0.01–47.62)
ANC (N = 626)		
0	**3.54 (1.92–6.53)**	0.51 (0.21–1.21)
1	**1.53 (0.79–2.96)**	0.48 (0.19–1.13)
2 to 3	**1.02 (0.61–1.72)**	0.68 (0.39–1.20)
≥ 4	1	1
Gestational age (N = 594)		
< 24 weeks	**148.36 (20.17–1091.30)**	**190.15 (22.62–1598.87)**
24–36 weeks	**126.11 (17.07–931.54)**	**167.48 (21.94–1278.32)**
≥ 37 weeks	1	1

cOR and aOR in bold are statistically significant

### Underlying causes for SMO and associated case fatality rates

The leading direct causes of several maternal outcomes, as shown in Table 4, are postpartum haemorrhage (accounting for 23.4% of all underlying causes), followed by uterine rupture (22.9%), abortion related complications (16.8%), and hypertensive disorders (8.9%). Malaria (laboratory confirmed) was the leading cause for indirect obstetric causes, accounting for 13.6% of all underlying causes. The CFR was high for women with hypertensive disorders (10.5%; CI 3.3–24.3), followed by severe postpartum haemorrhage (8.0%; CI 0.5–15.5) and malaria (6.9%; CI 2.3–16.1). During the study period, only one hospital (Rwamagana) had its own blood bank. Among the 49 cases with uterine rupture, 32 (65.3%) had a previous caesarean section and for 6 cases (12.2%) hysterectomy was performed.

**Table 4:** Underlying causes of severe maternal outcomes (near-miss and maternal deaths) and their associated CFR

Direct causes	MNM (*n* = 201)	MD (*n* = 13)	Total	CFR
Severe postpartum haemorrhage	46 (22.9%)	4 (30.8%)	50 (23.4%)	8.0% (0.5–15.5)
Ruptured uterus	46 (22.9%)	3 (23.1%)	49 (22.9%)	6.1% (0.6–12.8)
Severe complications of abortion	35 (17.4%)	1 (7.7%)	36 (16.8%)	2.9% (2.6–8.4)
Hypertensive disorders	17 (8.5%)	2 (15.4%)	19 (8.9%)	10.5% (3.3–24.3)
Puerperal sepsis	15 (7.5%)	0	15 (7%)	0
Abnormal/ectopic pregnancy	3 (1.5%)	0	3 (1.4%)	0
Severe intrapartum haemorrhage	1 (0.5%)	1 (7.7%)	2 (0.9%)	50%
Antepartum haemorrhage	1 (0.5%)	0	1 (0.5%)	0
Indirect causes				
Malaria (laboratory confirmed)	27 (13.4%)	2 (15.4%)	29 (13.6%)	6.9% (2.3–16.1)
Unknown causes of anaemia requiring blood transfusion	10 (5.0%)	0	10 (4.7)	0

Previous caesarean sections and anaemia were the two predominant contributory factors, accounting for 26.9% and 26.6%, respectively. In 42.1% of SMO cases any contributory factor was identified (not shown in the table).

Health providers used oxytocin as part of active management of third stage of labor in 94.9% of SMO cases and controls combined (Table 5). Among those who were diagnosed with severe PPH (58 cases and controls), about two-thirds (65.5%) received oxytocin only, while among the other third, three women received ergometrine and/or misoprostol in addition to oxytocin; in seven cases removal of retained placenta was performed in combination with oxytocin; six cases underwent hysterectomy (10.3%).

**Table 5:** Adherence to clinical standards for management of obstetric complications

Use of uterotonics for Prevention of postpartum haemorrhage	
Target population women giving birth at DH	N_1_ = 511^a^
Oxytocin	485 (94.9%)
Misoprostol	7 (1.4%)
All uterotonics	492 (96.3%)
Treatment of PPH	
Target population women severe PPH	N_2_ = 58
Oxytocin	38 (65.5%)
Oxytocin/Removal of retained placenta	7 (12.1%)
Oxytocin/Misoprostol	3 (5.2%)
Misoprostol	1 (1.7%)
Hysterectomy	6 (10.3%)
Use of anticonvulsants	
Target population women with severe (pre-) eclampsia	N_3_ = 19
Magnesium sulfate	18 (94.7%)
Diazepam	1 (5.3%)
Prevention of caesarean section /laparotomy related infection	
Target population undergoing Caesarean section/laparotomy	N_4_ = 300^b^
Prophylactic antibiotics	456 (98.6%)
Treatment of sepsis	
Target population women with sepsis	N_5_ = 15
Parenteral therapeutic antibiotics	15 (100%)

^a^511 cases among SMO and controls gave birth at district hospital

^b^249 cases of caesarean sections (Table 1) plus 51 cases of laparotomy

All severe (pre-) eclampsia cases received an anticonvulsant at the hospital, mostly magnesium sulphate (94.7%). Antibiotics were used for all women with an infection (15 cases) and almost all women who underwent a caesarean section or laparotomy received prophylactic antibiotics (98.6%). In total, 171 (80%) women of all SMO cases required blood transfusion; laparotomy was performed in 51 cases (23.8%).

## Discussion

This is the first prospective multicentre case control study combining maternal death and maternal near-miss in Rwandan district hospitals, where geographic access to emergency obstetric care is more of an issue than in Kigali capital city [9, 10]. Based on an analysis of SMO that occurred in four district hospitals, this study assessed the quality of care provided, using the WHO criteria adapted to the local context [14, 15].

The hospital based maternal mortality ratio was 233 (CI 110–360) per 100,000 live births. SMO.

and near-miss case ratios were relatively high at 38.4 (CI 33.4–43.4) and 36.0 (CI 31.1–40.9) per 1000 live births, respectively. Our study found a low mortality index (6.10) and a high maternal near-miss mortality ratio (15.5). Oxytocin was used for PPH prevention at 96.5% of all evaluated cases; magnesium sulphate as anticonvulsants in case of severe pre-eclampsia or eclampsia at 94.7%; and almost in all cases antibiotics were used in prophylaxis of sepsis in the event of a caesarean section or laparotomy, and in treatment of puerperal sepsis. Severe postpartum haemorrhage (23.4%), uterine rupture (22.9%), severe complications of abortion (16.8%), malaria (13.6%) and hypertensive disorders (8.9%) were the predominant causes of SMO. Case fatality for hypertensive disorders (eclampsia/pre eclampsia) was high in our settings at 10.5%. Being unmarried and developing a complication while gestational age was less than 36 weeks were identified as predictors for developing SMO and cases were associated with long stay at the hospital and stillbirth.

We found a hospital based maternal mortality ratio which corresponds with the 2015 DHS findings [4] and estimates in a recent UN report [1] which were 210 and 290 per 100,000 live births respectively. However, severe maternal outcome and near-miss case ratios were much higher than those found in a hospital-based study conducted in one of the tertiary hospitals in Kigali and in Musanze district hospital, which reported, SMO ratios of 11.0 and 24.8, and MNM ratios of 8 and 21.5 per 1000 live births, respectively [9, 12]. The near miss prevalence of our present study falls within the range of findings reported in the two systematic reviews of near-miss studies, one for Sub-Saharan Africa and the other for Africa as a whole, which found prevalence rates ranging from 1.1% to 10.1% and from 0.05 to 15.0%, respectively [17, 18]. Both ratios were high, though, compared to the WHO multi-country survey on maternal and newborn health, which found SMO and MNM ratios of 6.2 and 8.6 per 1000 live births respectively for high MMR countries, and 13.1 and 15.9 per 1000 live births for very high MMR countries, with overall rates of 8.3 and 9.9 per 1000 live births, respectively [19].The high ratios in our study may be explained by the fact that almost all women who deliver at district hospitals in Rwanda are referred, either because of high-risk pregnancy or complications that have occurred. However, they indicate a third phase delay [20]: either in making a diagnosis or deciding to refer the patient; or delays in the referral process at health centre level. Also, the threshold of blood transfusion (≥1 unit) used in our study, as per Haydom criteria, is much lower compared to the WHO criteria for near-miss (≥5 units), which may further explain the high ratios [15].

The combination of a low mortality index found in our study compared to other studies [17, 19] and high maternal near-miss mortality ratios compared to other settings [9, 12, 21–26], could be attributed, at least in part, by the frequent use of lifesaving interventions observed in our study. A high coverage of those interventions alone does not avoid the occurrence of SMO as shown in our study. Different studies have highlighted that high coverage of essential interventions is not sufficient to reduce maternal morbidity and mortality; they suggest that universal coverage of life-saving interventions needs to be matched with comprehensive emergency care and overall improvements in the quality of maternal health care [19, 27, 28]. Case fatality for hypertensive disorders in pregnancy and/or labour was also high in other studies, illustrating that better treatment of hypertension and starting induction of labour as soon as possible is needed to improve health outcomes [15, 21, 24, 29].

Except for marital status, other studies also identified age, educational level, parity, booking for ANC and gestational age as predictive factors for SMO [11, 22, 30, 31]; and stillbirth, long duration of admission, caesarean section, assisted vaginal delivery, birth asphyxia and low birth weight as associated factors [17, 32, 33].

While we used the four predictive characteristics identified in other studies as criteria for matching, we were unable to apply them simultaneously [11, 22, 30, 31]. This was due to the time limitation for the selection of the controls (maximum 48 h). Therefore, we selected controls that were similar to the cases for at least one of the four matching criteria; this is a limitation of the study. Although we did analyse the coverage of life saving interventions, we were not able to assess whether the interventions were implemented appropriately. Also, we used only SMO cases to determine the relatively CFRs, instead of all cases with particular obstetrical complications and this could explain the high rate found in our study as not all cases fitted the SMO criteria.

## Conclusions

Severe maternal outcomes are frequent. The high ratios of SMO and coverage of life saving interventions call for improvements in the quality of case management and follow up of pregnant women in order to reduce maternal morbidity and mortality. PPH, eclampsia and ruptured uterus are conditions that need particular attention as these are major causes of SMO and their case fatality rates are high.

Unmarried women and women with gestational age below 36 weeks are more likely to develop an SMO and this is associated with a longer stay at the hospital and with stillbirth. Surveillance of near miss events would be a useful addition to maternal death audits. Ideally, the two instruments should be integrated into routine monitoring and surveillance, not necessarily with the intention to examine all near-miss events, but focused on maternal conditions that are known to have the highest CFR, especially PPH, eclampsia and ruptured uterus. Increasing the coverage of life-saving interventions – such as using oxytocin in the management of third stage of labour, which is currently a policy in many countries and which is also recommended by WHO – is appropriate but insufficient. There is a need for improvement of quality of care at the level of district hospitals, through improved referral practices and certain types of infrastructure such as blood banks; this would go a long way in providing true comprehensive emergency obstetric care. Health centres will continue to refer women with obstetric complications to district hospitals, and although certain delays are unavoidable they should be minimised as much as possible. There is much to be gained from routine confidential enquiry into obstetric cases, including near-miss events, so as to learn from the way they are managed at the various levels of the referral chain.

## Additional file

Additional file 1: Table S1. Comparison table of WHO and Haydom criteria for near miss cases. (DOCX 18 kb)

## Abbreviations

CI: Confidence intervals
HMIS: Health management information system
ICU: Intensive care unit
MD: Maternal death
MDA: Maternal death audits
MDG: Millennium development goals
MI: Mortality index
MMR: Maternal mortality ratio
MNM: Maternal near-miss
MNMR: Maternal near-miss mortality ratio
RDHS: Rwanda demographic health surveys
SDG: Sustainable development goals
SMO: Severe maternal outcome
SMOR: Severe maternal outcome ratio
WHO: World Health Organization
