# Supplementary material for: Severe maternal outcomes and quality of care at district hospitals in Rwanda– a multicentre prospective case-control study
Source: BMC Pregnancy Childbirth. 2017 Nov 25;17:394. doi: 10.1186/s12884-017-1581-4 (PMC5702108; doi:10.1186/s12884-017-1581-4)
Supplement: Additional file 1: Table S1. — Comparison table of WHO and Haydom criteria for near miss cases. (DOCX 18 kb) [file 12884_2017_1581_MOESM1_ESM.docx]

**Table S1. Comparison of WHO and Haydom criteria for near miss cases**

| **Clinical criteria: WHO Haydom** | |
| --- | --- |
| -Acute cyanosis  -Gasping  -Respiratory rate >40 or <6/min  -Shock  -Oliguria non responsive to fluids or diuretics  - Failure to form clots  -Loss of consciousness lasting 12 hours  -Cardiac arrest  -Stroke  -Uncontrollable fit/total paralysis  -Jaundice in the presence of pre-eclampsia | -Acute cyanosis  -Gasping  -Respiratory rate >40 or <6/min  -Shock ^a^  -Oliguria non responsive to fluids or diuretics ^b^  - Failure to form clots ^c^  -Loss of consciousness lasting 12 hours ^d^  -Cardiac arrest ^e^  -Stroke ^f^  -Uncontrollable fit/total paralysis ^g^  -Jaundice in the presence of pre-eclampsia ^h^ |
| **Laboratory-based criteria** | |
| -Oxygen saturation <90% for 60 minutes  -PaO2/FiO2 <200 mmHg Lactate >5  -Creatinine 300 mmol/l or 3,5 mg/dl  -pH<7.1  -Acute thrombocytopenia (<50 000 platelets)  -Loss of consciousness AND the presence of glucose and ketoacids in urine  -Bilirubin>100 mmol/l or > 6,0 mg/dl | -Oxygen saturation <90% for 60 minutes  -Acute thrombocytopenia (<50 000 platelets) |
| **Management-based criteria** | |
| -Use of continuous vasoactive drugs  -Intubation and ventilation for 60 minutes not related to anaesthesia  -Hysterectomy following infection or haemorrhage  -Dialysis for acute renal failure  -Transfusion of ≥ 5 units of blood  -Cardio-pulmonary resuscitation (CPR) | -Admission to intensive Care Unit  - Intubation and ventilation for 60 minutes not related to anaesthesia  -Hysterectomy following infection or haemorrhage  -Transfusion of ≥ 1 unit of blood  -Cardio-pulmonary resuscitation (CPR) |
| **Severe maternal complications** | |
|  | -Eclampsia ^i^  -Sepsis or severe systemic infection ^j^  -Uterine rupture ^k^ |

a: Shock is defined as a persistent severe hypotension, defined as a systolic blood pressure < 90 mmHg for 60 min with a pulse rate of ≥ 120/min despite

aggressive fluid replacement (>2 liter).

b: Oliguria is defined as an urinary output < 30 ml/hour for 4 hours or < 400 ml/24 hr.

c: Failure to form clots is defined as the absence of clotting from the IV site after 7–10 minutes.

d: Unconsciousness/coma lasting > 12 hours is defined as a profound alteration of mental state that involves complete or near-complete lack of responsiveness to

external stimuli or Glasgow Coma Scale < 10.

e: Cardiac arrest is defined as loss of consciousness and absence of pulse or heart beat.

f: Stroke is defined as a neurological deficit of cerebrovascular cause that persists ≥ 24 hours, or is interrupted by death within 24 hours.

g: Uncontrollable fit is a condition in which the brain is in state of continuous seizure.

h: Pre-eclampsia: the presence of hypertension associated with proteinuria. Hypertension is defined as a blood pressure ≥ 140 mmHg (systolic) or ≥ 90 mmHg

(diastolic). Proteinuria is defined as excretion of ≥ 300 mg protein/24 hr or 300 mg protein/liter urine or ≥ 1+ on a dipstick.

i: Eclampsia is defined as the presence of hypertension associated with proteinuria and fits. Hypertension is defined as a blood pressure ≥ 140 mmHg (systolic)

or≥ 90 mmHg (diastolic). Proteinuria is defined as excretion of ≥ 300 mg protein/24 hr or 300 mg protein/liter urine or ≥ 1+ on a dipstick.

j: Sepsis is defined as a clinical sign of infection and 3 of the following: temp > 38°C or < 36°C, respiration rate > 20/min, pulse rate > 90/min, WBC >12.

k: Uterine rupture is defined as the complete rupture of a uterus (including peritoneum) with (partial) extrusion of the foetus during labour.
